# Supplementary material for: Effects of Soy-Based Infant Formula on Weight Gain and Neurodevelopment in an Autism Mouse Model
Source: Cells. 2022 Apr 15;11(8):1350. doi: 10.3390/cells11081350 (PMC9025435; doi:10.3390/cells11081350)
Supplement: Supplementary file 1 [file cells-11-01350-s001.zip › cells-1681104-supplementary.pdf]

cells MDPI

Supplementary Figures

Effects of Soy-Based Infant Formula on Weight Gain and Neurodevelopment in an Autism Mouse Model

Cara J. Westmark [1,2,*], Mikolaj J. Filon [1,3], Patricia Maina [1,4], Lauren I. Steinberg [1,3], Chrysanthy Ikonomidou [1], and Pamela R. Westmark [1]

1 Department of Neurology, University of Wisconsin, Madison, WI USA
2 Molecular Environmental Toxicology Center, University of Wisconsin, Madison, WI USA
3 Undergraduate Research Program, University of Wisconsin, Madison, WI USA
4 Molecular Environmental Toxicology Summer Research Opportunities Program, University of Wisconsin, Madison, WI USA

* Correspondence: westmark@wisc.edu; Tel.: 608-262-9730

Supplementary Figure S1: Body weights of juvenile WT and *Fmr1*^{KO} female and male mice in response to rodent diets. Mice were weighed on postnatal day 21 (P21) immediately prior to seizure testing: female WT/D07030301 (n=45), female WT/Purina 5015 (n=26), female *Fmr1*^{KO}/D07030301 (n=10), female *Fmr1*^{KO}/Purina 5015 (n=24), male WT/D07030301 (n=80), male WT/Purina 5015 (n=29), male *Fmr1*^{KO}/D07030301 (n=17), and male *Fmr1*^{KO}/Purina 5015 (n=32). Statistics were determined by 2-way ANOVA: females interaction F(1,101)=2.296, *p*=0.13; genotype F(1,101)=8.500, *p*=0.0044; diet F(1,101)=8.989, *p*<0.0034; males interaction F(1,154)=3.590, *p*=0.06; genotype F(1,154)=3.323, *p*=0.070; diet F(1,154)=18.49; *p*<0.0001; and Tukey's multiple comparison tests denoted by *p*<0.05 (*) and *p*<0.01 (**).

Supplementary Figure S2: Body weights of adult WT female and male mice in response to rodent diets. C57BL/6J mice were maintained on Purina 5015 for 1 month and then transferred to D07030301 or left on the Purina 5015 for an additional 1 month. Mice were weighed at 2 months of age immediately prior to PTZ-induced seizure testing: female D07030301 (n=26), female Purina 5015 (n=20), male D07030301 (n=39) and male Purina 5015 (n=20). Statistics were determined by Student's t-tests: females (*p*=0.074) and males (*p*=0.067).

Supplementary Figure S3: EchoMRI measurements of total body weight, fat mass, lean mass, total water, and free water in female *Fmr1*^{HET} and *Fmr1*^{KO} and male WT and *Fmr1*^{KO} mice as a function of Teklad 2019, CIF and SIF diets at age P62. Female cohorts included: *Fmr1*^{HET}/Teklad 2019 (n=4), *Fmr1*^{HET}/CIF (n=5), *Fmr1*^{HET}/SIF (n=6), *Fmr1*^{KO}/Teklad 2019 (n=6), *Fmr1*^{KO}/CIF (n=7) and *Fmr1*^{KO}/SIF (n=6). Male cohorts included: WT/Teklad 2019 (n=6), WT/CIF (n=6), WT/SIF (n=4), *Fmr1*^{KO}/Teklad 2019 (n=6), *Fmr1*^{KO}/CIF (n=5) and *Fmr1*^{KO}/SIF (n=7). Statistics were determined by two-way ANOVA. Tukey's multiple comparison tests denoted by *p*<0.05 (*), *p*<0.01 (**), *p*<0.001 (***), and *p*<0.0001 (****).

Supplementary Figure S4: EchoMRI measurements of total body weight, fat mass, lean mass, total water, and free water in female *Fmr1*^{HET} and *Fmr1*^{KO} and male WT and *Fmr1*^{KO} mice as a function of Teklad 2019, CIF and SIF diets at age P76. Female cohorts included: *Fmr1*^{HET}/Teklad 2019 (n=4), *Fmr1*^{HET}/CIF (n=4), *Fmr1*^{HET}/SIF (n=6), *Fmr1*^{KO}/Teklad 2019 (n=6), *Fmr1*^{KO}/CIF (n=7) and *Fmr1*^{KO}/SIF (n=6). Male cohorts included: WT/Teklad 2019 (n=6), WT/CIF (n=6), WT/SIF (n=4), *Fmr1*^{KO}/Teklad 2019 (n=6), *Fmr1*^{KO}/CIF (n=5) and *Fmr1*^{KO}/SIF (n=7). Statistics were determined by two-way ANOVA. Tukey's multiple comparison tests denoted by *p*<0.05 (*), *p*<0.01 (**), and *p*<0.001 (***).

Supplementary Figure S5: Infant feeding during the first 3 months of life is associated with growth metrics at 12 months of age. (A) Cohorts included females fed breast milk (n=218), cow milk formula (n=240) and soy-based infant formula (n=15) and males fed breast milk (n=201), cow milk formula (n=215) and soy-based infant formula (n=21). Statistics were determined by two-way ANOVA body weight interaction F(2,706)=0.4863, *P*=0.61; diet F(2,706)=17.37, *P*<0.0001; sex F(1,706)=25.00, *P*<0.0001; height interaction F(2,512)=0.1625, *P*=0.85; diet F(2,512)=2.697, *P*=0.0683; sex F(1,512)=4.169, *P*=0.0417; and BMI interaction F(2,511)=0.06707, *P*=0.94; diet F(2,511)=6.485, *P*=0.0017; sex F(1,511)=1.609, *P*=0.2052.

Supplementary Figure S6: Infant feeding during the first 3 months of life is associated with growth metrics at 6 years of age. (A) Cohorts included females fed breast milk (n=218), cow milk formula (n=240) and soy-based infant formula (n=15) and males fed breast milk (n=201), cow milk formula (n=215) and soy-based infant formula (n=21). Statistics were determined by two-way ANOVA body weight interaction F(2,870)=0.9167, *P*=0.40; diet F(2,870)=10.66, *P*<0.0001; sex F(1,870)=1.075, *P*=0.30; height interaction F(2,848)=0.4322, *P*=0.65; diet F(2,848)=7.895, *P*=0.4544; sex F(1,848)=2.45, P=0.12; and BMI interaction F(2,826)=1.163, *P*=0.31; diet F(2,826)=7.812, *P*=0.0004; sex F(1,826)=3.08E-6, *P*=0.999.

Supplementary Figure S1: Juvenile Mice

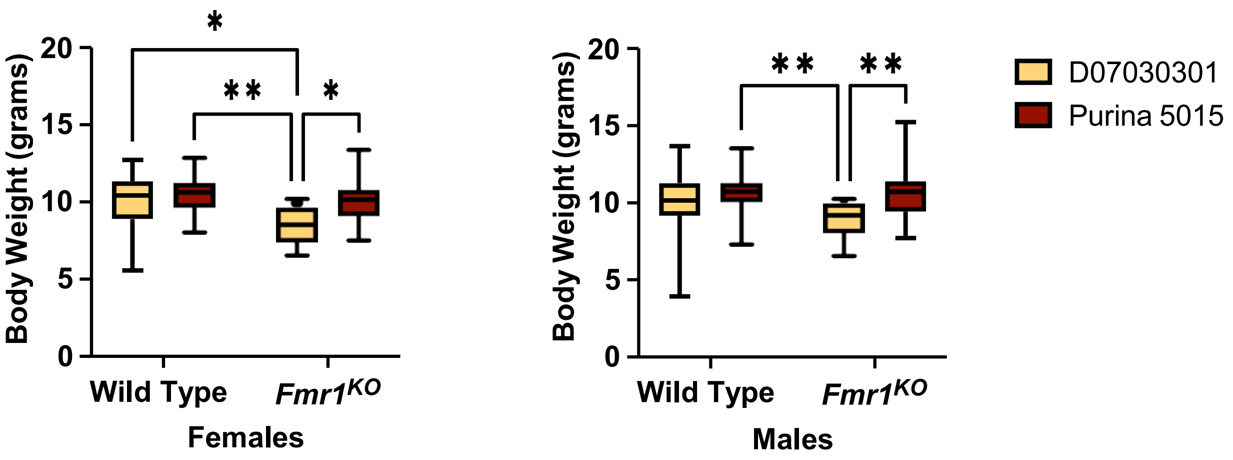

Supplementary Figure S2

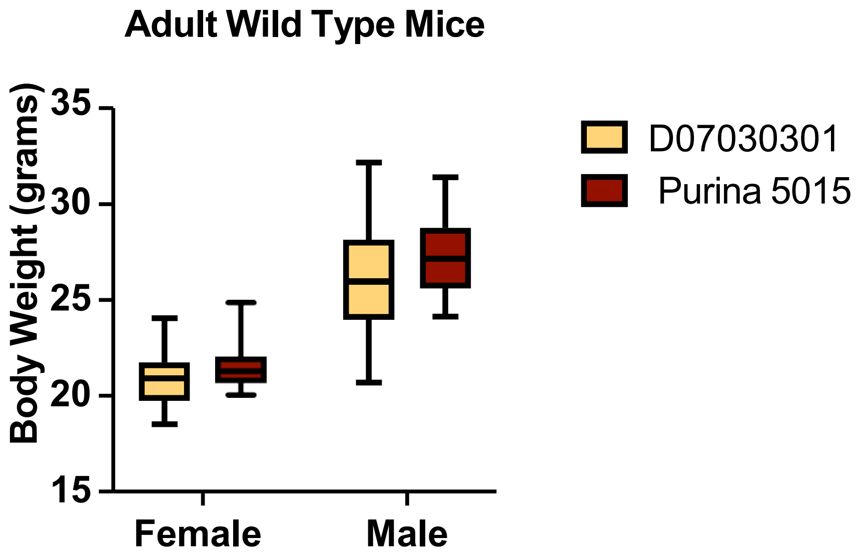

Supplementary Figure S3

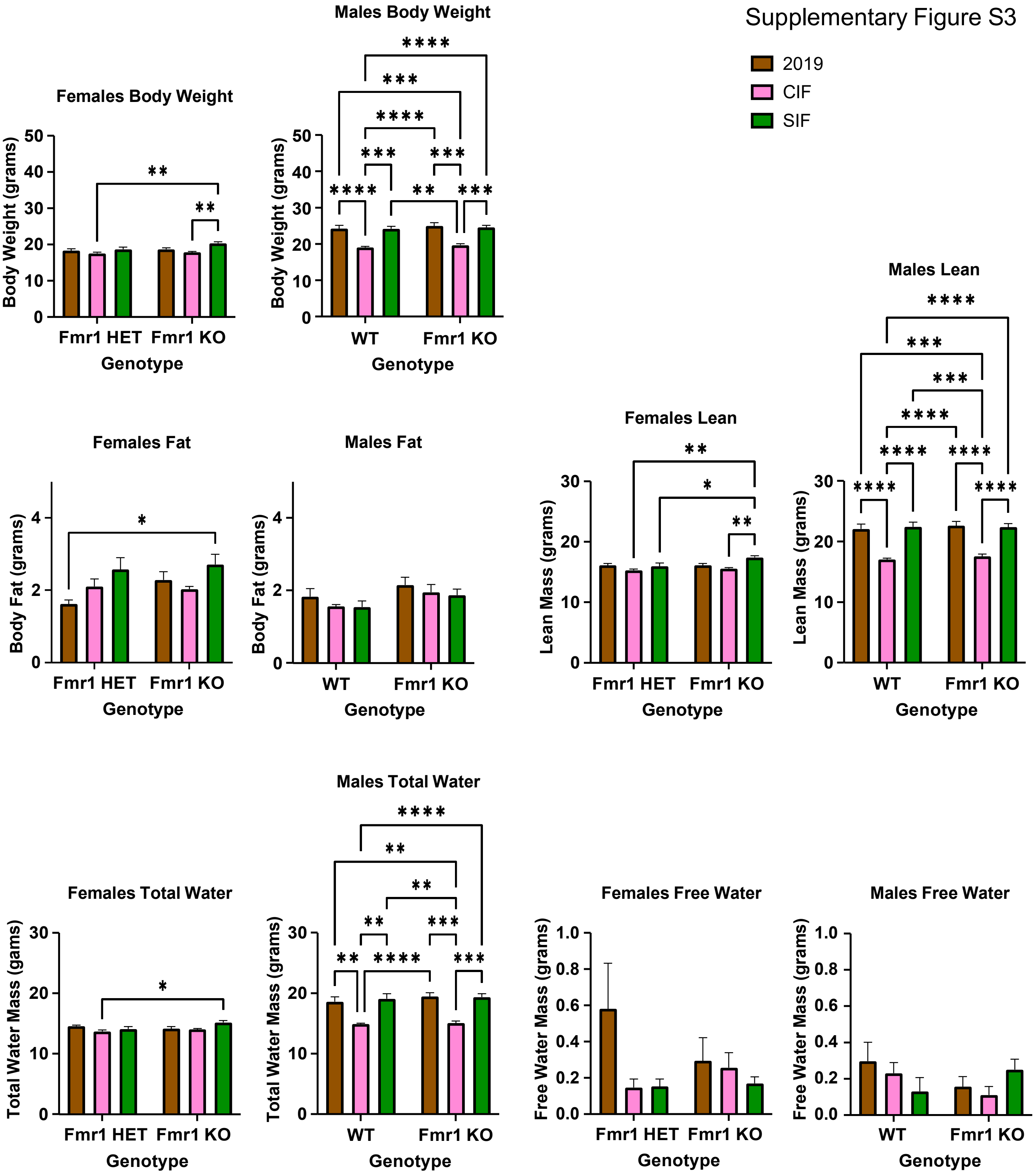

Supplementary Figure S4

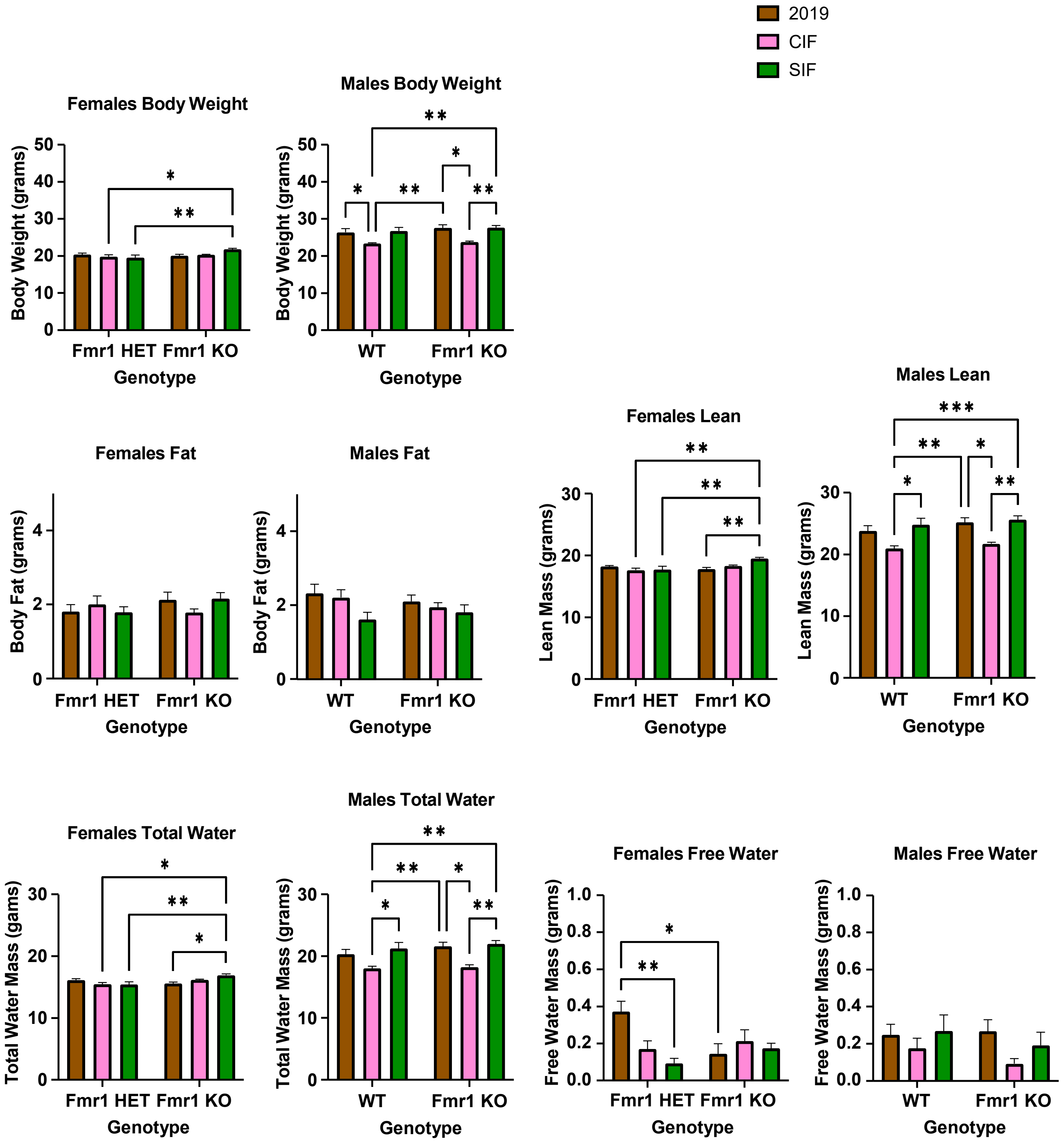

Supplementary Figure S5

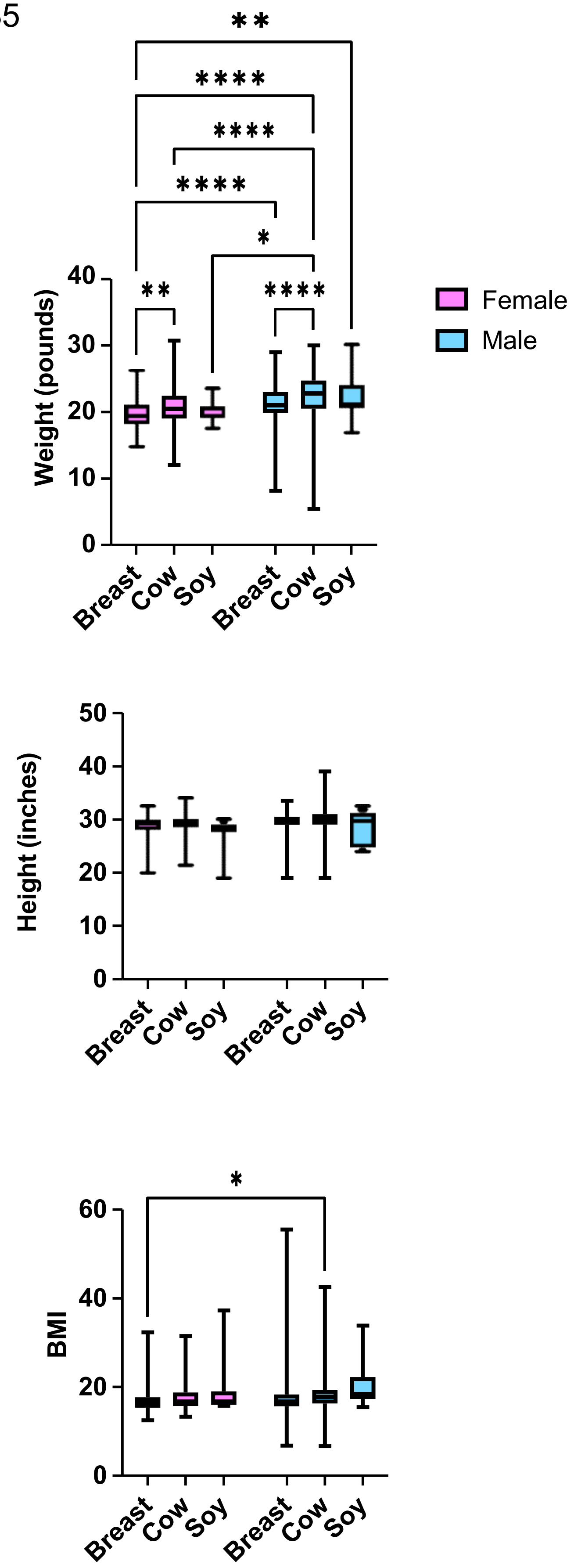

Supplementary Figure S6

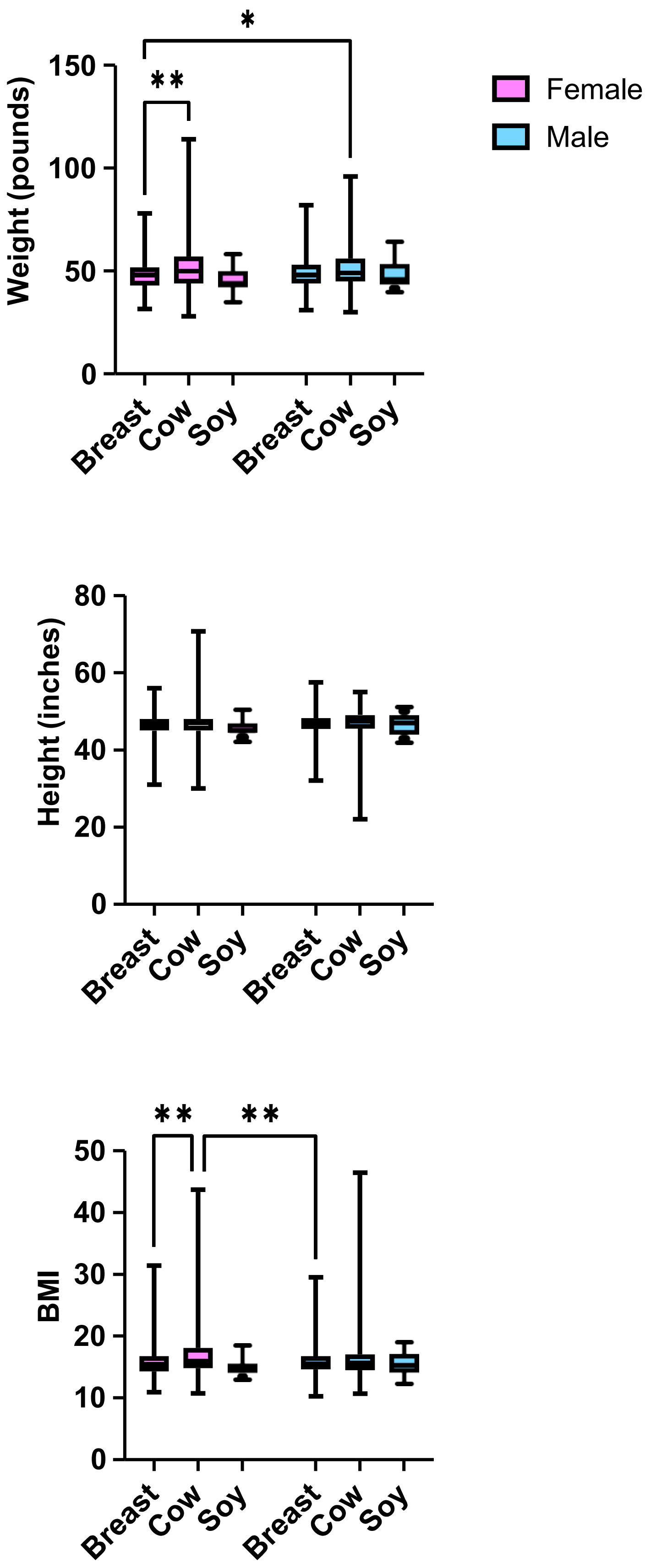

cells MDPI

Supplementary Tables

Effects of Soy-Based Infant Formula on Weight Gain and Neurodevelopment in an Autism Mouse Model

Cara J. Westmark [1,2,*], Mikolaj J. Filon [1,3], Patricia Maina [1,4], Lauren I. Steinberg [1,3], Chrysanthy Ikonomidou [1], and Pamela R. Westmark [1]

1 Department of Neurology, University of Wisconsin, Madison, WI USA
2 Molecular Environmental Toxicology Center, University of Wisconsin, Madison, WI USA
3 Undergraduate Research Program, University of Wisconsin, Madison, WI USA
4 Molecular Environmental Toxicology Summer Research Opportunities Program, University of Wisconsin, Madison, WI USA

* Correspondence: westmark@wisc.edu; Tel.: 608-262-9730

Supplementary Table S1: Composition of Purified Ingredient Diets.

	**D07030301<br>Modified AIN-76A**	**D10012G<br>(AIN-93G)**
Protein	18%	20%
Carbohydrate	55.5%	64%
Fat	11.6%	7%
**Ingredient**	**grams**	**grams**
Casein	180	200
L-Cystine	3	3
Corn Starch	431	397
Maltodextrin 10	125	132
Inulin	25	0
Sucrose	0	100
Cellulose, BW200	75	50
Soybean Oil	118	70
t-Butylhydroquinone	0	0.014
Choline Bitartrate	2	2.5
**Vitamins & Minerals**		
Vitamin A, Acetate (500,000 IU/gm)	4000 IU	4000 IU
Vitamin D3 (100,000 IU/gm)	1000 IU	1000 IU
Vitamin E Acetate (500 IU/gm)	50 IU	75 IU
Menadione Sodium Bisulfite<br>(62.5% Menadione)	0.5 mg	0 mg
Phylloquinone	0 mg	0.75 mg
Biotin, 1.0%	0.2 mg	0.2 mg
Cyanocobalamin, 0.1%	10 mcg	25 mcg
Folic Acid	2 mg	2 mg
Nicotinic Acid	30 mg	30 mg
Calcium Pantothenate	16 mg	16 mg
Pyridoxine-HCl	7 mg	7 mg
Riboflavin	6 mg	6 mg
Thiamin HCl	6 mg	6 mg
Calcium Phosphate, Dibasic<br>(29.5% Ca, 22.8% P)	6 g	0 g
Calcium Carbonate (39.3%, 40.0% Ca)	3.0 g	5.0 g
Potassium Phosphate, Monobasic<br>(28.7% K, 22.8%P)	0 g	1.56 g
Potassium Citrate · 1 H20 (36.2% K)	6.0 g	3.6 g
Potassium Sulfate (44.9% K, 18.4% S)	0 g	0.3 g
Magnesium Oxide (60.3 Mg)	0.5 g	0.5 g
Magnesium Sulfate · 7 H20<br>(9.87% Mg, 13.0% S)	0.33 g	0 g
Sodium Chloride (39.3% Na, 60.7% Cl)	1.0 g / 1.6 g	1.0 g, 1.6 g
Cupric Carbonate (57.5% Cu)	6.0 mg	6.0 mg
Potassium Iodate (59.3% I)	0.2 mg	0.2 mg
Ferric Citrate (21.2 %, 17.4% Fe)	45 mg	37 mg
Manganese Carbonate (47.8% Mn)	59 mg	10.5 mg
Sodium Selenate (45.7%, 41.8% Se)	0.16	0.2 mg
Zinc Carbonate (52.1%, 25.1% Zn)	29 mg	30 mg
Chromium K Sulfate · 12 H20 (10.4% Cr)	2.0 mg	962.5 mcg
Ammonium Molybdate · 4 H20<br>(54.3% Mo)	1.6 mg	151.1 mcg
Sodium Fluoride (45.2% F)	0.9 mg	1 mg

**Supplementary Table S2: Composition of Casein- and Soy-Based Infant Formula Diets Formulated for Mice.**		
**Ingredient**	**D14010401 / SIF Enfamil ProSobee**	**D14010402 / CIF Enfamil Premium**
	**grams**	**grams**
Enfamil ProSobee	1000	0
Enfamil Premium	0	1000
DL-Methionine	3	3
DiCalcium Phosphate	0	4
Calcium Carbonate	1.5	2
Magnesium Oxide	0.2	0.2
Copper Carbonate	0.005	0.005
Manganous Carbonate	0.03	0.03
Ammonium Molybdate	0.0004	0.0004
Menadione Sodium Bisulfite (62.5% Menadione)	0.001	0.001
Biotin	0.0001	0.0001
Pyridoxine HCL	0.006	0.006
Riboflavin	0.004	0.004
Thiamin HCL (80% Thiamin)	0.002	0.002
Choline Bitartrate	2	2
FD&C Blue Dye #1	0.05	0
FD&C Red Dye #40	0	0.05

**Supplementary Table S3: Micronutrient Content of Infant Formula Diets Formulated forMice.**			
**Micronutrient (per kg diet)**	**NRC Recommended[1]**	**SIF D14010401 Enfamil ProSobee**	**CIF D14010402 Enfamil Premium**
Vitamin A (IU)	2400 IU	16190 IU	15950 IU
Vitamin D (IU)	1000 IU	3278 IU	3194 IU
Vitamin E (IU)	32 IU	109 IU	109 IU
Vitamin K (mg)	1 mg	1.1 mg	1.1 mg
Biotin (mg)	0.2 mg	0.21 mg	0.21 mg
B12 (mg)	0.01 mg	0.02 mg	0.02 mg
Folic Acid (mg)	0.5 mg	0.9 mg	0.9 mg
Niacin (mg)	15 mg	53.6 mg	53.4 mg
Pantothenate (Ca) (mg)	16 mg	26.8 mg	26.7 mg
B6 (Pyridoxine-HCl) (mg)	8 mg	8.9 mg	8.9 mg
Riboflavin (mg)	7 mg	8.9 mg	11.4 mg
Thiamin-HCl (mg)	5 mg	5.9 mg	5.8 mg
Methionine (g)	5 g	3.0 g	3.0 g
Sodium (g)	0.5 g	2.0 g	1.5 g
Chloride (g)	0.5 g	4.3 g	3.4 g
Calcium (g)	5 g	6.2 g	6.1 g
Phosphorus (g)	3 g	3.7 g	3.2 g
Potassium (g)	2 g	6.5 g	5.7 g
Magnesium (g)	0.5 g	0.5 g	0.5 g
Sulfur (g)	ND	0.0 g	0.0 g
Chromium mg)	ND	0.0 mg	0.0 mg
Copper (mg)	6 mg	6.9 mg	6.8 mg
Fluoride (mg)	ND	0.0 mg	0.0 mg
Iodine (mg)	0.15 mg	0.8 mg	0.8 mg
Iron (mg)	35 mg	99.3 mg	98.9 mg
Manganese (mg)	10 mg	15.6 mg	15.0 mg
Molybdenum (mg)	0.15 mg	0.22 mg	0.21 mg
Selenium (mg)	0.15 mg	0.15 mg	0.15 mg
Zinc (mg)	10 mg	64.6 mg	53.4 mg
Choline Bitartrate (g)	2 g	3.3 g	3.3 g

[1]NRC Guidelines as described in Nutrient Requirements of Laboratory Animals, 4th Revised Edition, 1995. Subcommittee on Laboratory Animal Nutrition, Board on Agriculture, National Research Council, National Academy Press, Washington, D.C.

**Supplementary Table S 4: Body Weights in JuvenileMice Dependent on Infant Formula Diets.**			
**Strain / Gender**	**Diet**	**n**	**Weight (g)**
WT / Female	CIF	0	ND
	SIF	3	8.07 ± 0.23
WT / Male	CIF	10	7.75 ± 0.35
	SIF	0	ND
Fmr1^{KO} / Female	CIF	5	7.52 ± 0.47
	SIF	5	6.70 ± 0.35
Fmr1^{KO} / Male	CIF	9	7.49 ± 0.25
	SIF	7	6.73 ± 0.53

Supplementary Table S5: Diet Dependent Differential Expression of RayBiotech Array 4 Proteins in WT and *Fmr1*^{KO} Plasma.									
	WT (pg/mL, SD)			Fmr1KO (pg/mL, SD)			2-Way ANOVA, F (*P*)		
**Protein**	**2019**	**CIF**	**SIF**	**2019**	**CIF**	**SIF**	**Interaction**	**Diet**	**Genotype**
Axl	6,030 (862)	12,200 (2,660)	10,600 (2,560)	8,020 (1,170)	9,600 (1,280)	7,440 (1,050)	3.89 (≤0.05)	7.31 (≤0.01)	2.31 (ns)
CD30	175 (22.1)	237 (10.4)	254 (46.7)	182 (35.5)	250 (20.8)	206 (30.9)	1.88 (ns)	7.81 (≤0.01)	0.43 (ns)
CXCL 16	505 (89.1)	475 (6.2)	470 (43.7)	533 (53.1)	432 (35.4)	408 (44.9)	1.27 (ns)	4.09 (≤0.05)	1.11 (ns)
EGF	52.5 (12.6)	46.1 (5.9)	45.1 (3.2)	36.7 (2.2)	43.6 (6.3)	38.2 (6.0)	1.45 (ns)	0.40 (ns)	6.70 (≤0.03)
Fractalkine	4,130 (708)	2,820 (1,980)	4,090 (171)	3,530 (555)	3,160 (456)	3,470 (895)	0.47 (ns)	1.39 (ns)	0.40 (ns)
IGFBP-2	7,520 (4,670)	28,800 (15,100)	9,520 (4,210)	21,000 (11,570)	8,990 (2,370)	11,900 (5,130)	5.97 (≤0.02)	1.40 (ns)	0.11 (ns)
IGFBP-5	10,500 (1,730)	11,900 (1,820)	11,500 (1,430)	13,800 (2,490)	8,710 (1,400)	12,000 (1,500)	5.08 (≤0.03)	1.81 (ns)	0.060 (ns)
IL-2 Ra	366 (27.0)	522 (29.6)	509 (126)	351 (69.9)	622 (202)	414 (31.0)	1.35 (ns)	6.40 (≤0.02)	0.0047 (ns)
IL-20	177 (93.9)	103 (69.0)	252 (192)	233 (183)	608 (581)	597 (667)	0.54 (ns)	0.52 (ns)	2.84 (ns)
MDC	169 (17.6)	199 (69.7)	229 (90.4)	184 (67.1)	159 (25.6)	142 (24.3)	1.23 (ns)	0.041 (ns)	1.97 (ns)
MIP-3a	35.9 (32.1)	73.4 (64.6)	24.0 (30.6)	7.5 (2.9)	118 (141)	21.8 (25.2)	0.46 (ns)	2.43 (ns)	0.022 (ns)
OPN	29,600 (5,070)	32,800 (9,320)	27,400 (6,620)	32,100 (4,860)	32,000 (7,880)	33,100 (5,060)	0.36 (ns)	0.17 (ns)	0.61 (ns)
OPG	928 (169)	1,450 (696)	1,660 (1,110)	903 (419)	929 (296)	636 (170)	1.10 (ns)	0.39 (ns)	3.63 (ns)
Prolactin	1,640 (508)	2,290 (1,960)	2,390 (2,070)	1,890 (1,150)	6,810 (726)	4,800 (3,140)	2.04 (ns)	3.59 (ns)	7.70 (≤0.02)
Pro-MMP-9	3,130 (1,760)	3,300 (1,240)	2,620 (2,700)	3,490 (3,870)	2,440 (752)	1,620 (852)	0.18 (ns)	0.46 (ns)	0.24 (ns)
VEGF	88.5 (12.4)	52.1 (39.0)	36.3 (39.4)	67.5 (7.1)	213 (198)	50.4 (31.0)	1.93 (ns)	1.68 (ns)	1.64 (ns)

Supplementary Table S6: Diet Dependent Differential Expression of RayBiotech Array 5 Proteins in WT and *Fmr1^{KO}* Plasma.

Protein	WT (pg/mL, SD)			Fmr1KO (pg/mL, SD)			2-Way ANOVA, F (*P*)		
	2019	CIF	SIF	2019	CIF	SIF	Interaction	Diet	Genotype
bFGF	83.9 (2.31)	50.0 (31.4)	65.5 (20.7)	63.3 (25.2)	90.8 (14.1)	98.2 (12.4)	4.16 (≤0.05)	0.52 (ns)	3.49 (ns)
BLC	254 (67.2)	221 (34.8)	180 (30.8)	122 (17.0)	161 (26.4)	161 (34.6)	3.33 (ns)	0.50 (ns)	15.1 (≤0.00)
Eotaxin	554 (33.3)	544 (9.0)	629 (95.3)	590 (25.0)	617 (172)	641 (76.1)	0.18 (ns)	0.91 (ns)	0.95 (ns)
Eotaxin-2	60.6 (12.9)	76.6 (27.1)	60.6 (24.2)	60.9 (17.0)	40.6 (9.2)	51.0 (12.2)	1.58 (ns)	0.11 (ns)	3.06 (ns)
ICAM-1	344 (312)	2090 (902)	568 (37.1)	985 (588)	564 (281)	516 (116)	8.16 (≤0.01)	4.75 (≤0.03)	1.95 (ns)
IL-12p40	53.7 (42.6)	40.3 (20.3)	96.7 (117)	63.2 (10.2)	82.9 (97.4)	63.0 (39.1)	0.49 (ns)	0.18 (ns)	0.038 (ns)
Leptin	375 (334)	241 (216)	2130 (732)	293 (232)	1210 (958)	1160 (1360)	2.41 (ns)	4.64 (≤0.04)	0.0059 (ns)
MCP-5	17.3 (2.74)	18.7 (4.1)	68.8 (86.7)	23.3 (15.5)	23.9 (3.8)	16.7 (5.6)	1.28 (ns)	0.74 (ns)	0.64 (ns)
PF4	2380 (157)	2850 (509)	2300 (336)	2370 (578)	2430 (355)	2680 (379)	1.44 (ns)	0.64 (ns)	0.0075 (ns)

Supplementary Table S7: Diet Dependent Differential Expression of RayBiotech Array 6 Proteins in WT and *Fmr1*^{KO} Plasma.									
	WT (pg/mL, SD)			Fmr1KO (pg/mL, SD)			2-Way ANOVA, F (*P*)		
**Protein**	**2019**	**CIF**	**SIF**	**2019**	**CIF**	**SIF**	**Interaction**	**Diet**	**Genotype**
CD40L	1,310 (366)	1,350 (610)	1,270 (563)	1,610 (244)	1,910 (1,390)	803 (671)	0.79 (ns)	1.03 (ns)	0.14 (ns)
Dkk-1	4,070 (957)	3,580 (790)	3,610 (1,250)	4,380 (991)	4,360 (1,840)	3,110 (1,530)	0.38 (ns)	0.72 (ns)	0.11 (ns)
Endoglin	462 (135)	378 (170)	416 (257)	398 (117)	267 (297)	254 (122)	0.094 (ns)	0.54 (ns)	1.48 (ns)
Fcg RIIB	3,940 (1,180)	4,130 (675)	3,910 (621)	3,260 (918)	3,570 (975)	3,080 (736)	0.036 (ns)	0.26 (ns)	2.81 (ns)
Fit-3L	2,250 (181)	2,100 (296)	2,100 (251)	1,860 (113)	2,230 (184)	1,990 (184)	2.31 (ns)	0.60 (ns)	1.56 (ns)
Galectin-1	8,880 (1,320)	6,780 (3,350)	7,740 (4,460)	10,300 (3,230)	7,560 (3,900)	5,210 (2,730)	0.61 (ns)	1.46 (ns)	0.0050 (ns)
HAI-1	1,100 (358)	2,840 (1,440)	2,180 (1,050)	1,420 (679)	1,360 (360)	1,220 (326)	1.93 (ns)	1.59 (ns)	3.37 (ns)
HGF R	2,120 (494)	3,890 (1,050)	2,470 (1,530)	5,540 (3,280)	1,820 (910)	2,040 (1,340)	4.19 (≤0.05)	1.33 (ns)	0.15 (ns)
IL-1 R4	318 (84.1)	UL	UL	321 (48.4)	UL	UL	0.83 (0.46)	5.56 (≤0.02)	0.11 (0.75)
IL-3 Rb	1,210 (285)	UL	UL	1,250 (105)	UL	UL	0.020 (ns)	0.65 (ns)	0.00020 (ns)
JAM-A	1,210 (309)	1,600 (234)	1,290 (126)	1,020 (266)	1, 320 (122)	871 (338)	0.33 (ns)	4.33 (≤0.04)	6.48 (≤0.03)
Leptin R	599 (175)	2,180 (1,160)	1,580 (795)	628 (315)	899 (151)	1,180 (554)	1.65 (ns)	3.62 (ns)	3.36 (ns)
MadCAM-1	367 (215)	1,040 (452)	700 (204)	733 (683)	519 (99.0)	731 (258)	2.16 (ns)	0.60 (ns)	0.055 (ns)
MFG-E8	1,490 (1,040)	2,790 (171)	643 (228)	1,580 (969)	585 (89.3)	1,780 (1,360)	6.64 (≤0.02)	0.54 (ns)	0.72 (ns)
Neprilysin	1,040 (345)	1,140 (996)	4,550 (1,820)	899 (222)	969 (208)	2,200 (780)	2.82 (ns)	13.1 (≤0.00)	4.15 (ns)
RAGE	2,440 (1,300)	984 (1,220)	2,440 (1,860)	2,950 (837)	1,210 (1,640)	911 (878)	1.02 (ns)	2.18 (ns)	0.17 (ns)
TROY	618 (90.2)	551 (59.5)	605 (188)	599 (21.0)	530 (246)	381 (247)	0.74 (ns)	0.72 (ns)	1.24 (ns)
VEGF R1	255 (103)	154 (60.9)	246 (204)	260 (170)	275 (224)	77.4 (134)	1.25 (ns)	0.54 (ns)	0.036 (ns)

Supplementary Table S8: Diet Dependent Differential Expression of RayBiotech Array 7 Proteins in WT and *Fmr1*^{KO} Plasma.

	WT (pg/mL, SD)			Fmr1KO (pg/mL, SD)			2-Way ANOVA, F (*P*)		
**Protein**	**2019**	**CIF**	**SIF**	**2019**	**CIF**	**SIF**	**Interaction**	**Diet**	**Genotype**
B7-1	403 (66.2)	597 (92.5)	822 (104)	216 (34.3)	857 (523)	471 (91.6)	2.93 (ns)	5.76 (≤0.02)	0.76 (ns)
BAFF R	362 (46.9)	304 (151)	206 (148)	200 (134)	318 (258)	338 (172)	1.22 (ns)	0.093 (ns)	0.0048 (ns)
BTC	81.2 (62.5)	141 (74.3)	166 (92.0)	38.3 (21.5)	94.6 (53.7)	164 (100)	0.18 (ns)	3.20 (ns)	0.80 (ns)
C5a	710 (68.9)	756 (138)	733 (147)	646 (144)	709 (110)	767 (313)	0.14 (ns)	0.29 (ns)	0.10 (ns)
CCL6	2,410 (687)	2,380 (894)	2,880 (1,190)	2,400 (619)	2,970 (66.3)	2,660 (553)	0.47 (ns)	0.38 (ns)	0.12 (ns)
CD6	224 (191)	427 (44.6)	530 (247)	264 (146)	877 (414)	567 (398)	1.13 (ns)	3.59 (ns)	1.85 (ns)
Chemerin	35,700 (14,400)	76,000 (16,100)	62,100 (15,200)	51,600 (16,600)	67,300 (29,700)	43,600 (15,200)	1.36 (ns)	3.52 (ns)	0.18 (ns)
DAN	265 (152)	365 (219)	547 (180)	112 (96.7)	329 (253)	266 (222)	0.60 (ns)	2.02 (ns)	2.93 (ns)
DLL4	901 (1,550)	3,660 (3,630)	3,430 (2,310)	4.5 (7.8)	2,680 (2,130)	6,420 (5,510)	0.83 (ns)	3.28 (ns)	0.067 (ns)
Endocan	985 (609)	743 (631)	1,510 (561)	71.7 (124)	1,970 (1,920)	450 (738)	2.77 (ns)	1.16 (ns)	0.31 (ns)
Fetuin A	68,700 (90,000)	29,000 (22,500)	37,500 (47,400)	2,680 (592)	25,800 (18,300)	18,100 (18,900)	0.83 (ns)	0.068 (ns)	2.04 (ns)
IL-33	157 (74.5)	80.9 (80.7)	150 (138)	63.7 (73.2)	182 (142)	146 (129)	1.16 (ns)	0.18 (ns)	0.00059 (ns)
IL-7 Ra	1,580 (922)	1,260 (728)	531 (257)	164 (266)	1,500 (1,610)	1,460 (1,250)	2.31 (ns)	0.45 (ns)	0.032 (ns)
Limitin	47.4 (47.7)	59.5 (19.6)	71.0 (18.9)	36.8 (43.1)	55.8 (44.4)	67.5 (67.1)	0.013 (ns)	0.59 (ns)	0.084 (ns)
Lipocalin-2	32,800 (32,800)	43,300 (27,300)	51,200 (29,400)	8,740 (2,800)	46,000 (31,200)	76,300 (22,400)	1.31 (ns)	4.01 (≤0.05)	0.01 (ns)
Marapsin	344 (369)	384 (325)	385 (284)	76.8 (133)	223 (230)	531 (603)	0.55 (ns)	0.74 (ns)	0.32 (ns)
Nope	15,500 (1,940)	14,800 (2,800)	15,800 (2,470)	12,600 (1,640)	13,200 (1,260)	15,100 (2,230)	0.41 (ns)	0.90 (ns)	3.01 (ns)
NOV	6,530 (1,290)	8,600 (2,070)	7,340 (1,050)	6,390 (3,380)	8,680 (2,400)	7,020 (3,950)	0.0091 (ns)	1.12 (ns)	0.011 (ns)
Osteoactivin	1,890 (355)	2,610 (1,690)	2,670 (473)	2,380 (200)	2,950 (526)	3,830 (914)	0.39 (ns)	2.59 (ns)	2.73 (ns)
OX40 Ligand	105 (56.5)	55.5 (32.0)	93.5 (27.0)	22.2 (20.4)	92.7 (61.2)	72.5 (40.0)	3.03 (ns)	0.32 (ns)	1.24 (ns)
P-Cadherin	1,000 (324)	1,250 (261)	1,260 (520)	924 (71.5)	1,430 (530)	1,950 (271)	1.70 (ns)	4.68 (≤0.04)	2.36 (ns)
Renin 1	13,900 (1,130)	19,500 (7,180)	16,700 (2,490)	10,300 (2,390)	23,500 (6,140)	20,300 (9,750)	0.83 (ns)	4.20 (≤0.05)	0.24 (ns)
Testican 3	671 (502)	419 (192)	150 (51.2)	52.3 (46.3)	1,460 (1,880)	1,470 (1,430)	1.68 (ns)	0.56 (ns)	1.55 (ns)
TIM-1	849 (453)	1,090 (523)	998 (299)	833 (301)	992 (662)	1,250 (1,000)	0.14 (ns)	0.36 (ns)	0.027 (ns)
TRAIL	338 (165)	445 (597)	2,910 (4,560)	52,400 (90,700)	224 (203)	643 (803)	1.04 (ns)	0.93 (ns)	0.89 (ns)
Tryptase E	978 (401)	1,370 (419)	1,470 (309)	321 (150)	813 (856)	1,140 (706)	0.15 (ns)	2.39 (ns)	4.24 (ns)

Supplementary Table S9: Diet Dependent Differential Expression of RayBiotech Array 8 Proteins in WT and *Fmr1*^{KO} Plasma.

Protein	WT (pg/mL, SD)			Fmr1KO (pg/mL, SD)			2-Way ANOVA, F (*P*)		
	2019	CIF	SIF	2019	CIF	SIF	Interaction	Diet	Genotype
6Ckine	461 (114)	207 (107)	293 (169)	141 (92.2)	253 (68.4)	257 (44.9)	4.87 (≤0.03)	0.68 (ns)	4.23 (ns)
Adiponectin	5,050 (489)	4,910 (1,760)	4,380 (2,720)	5,530 (1,370)	4,620 (1,950)	4,110 (1,680)	0.090 (ns)	0.51 (ns)	0.0010 (ns)
ANGPTL3	115,000 (30,800)	122,000 (14,400)	115,000 (17,600)	129,000 (20,800)	95,300 (41,600)	87,100 (17,700)	1.30 (ns)	1.03 (ns)	1.25 (ns)
CCL28	395 (54.0)	297 (158)	389 (50.5)	335 (111)	447 (69.9)	523 (203)	1.38 (ns)	1.04 (ns)	1.69 (ns)
CD36	653 (49.2)	1,570 (117)	662 (522)	560 (134)	1,270 (471)	733 (720)	0.30 (ns)	6.83 (≤0.01)	0.30 (ns)
Chordin	386 (77.0)	245 (137)	299 (139)	419 (91.5)	378 (138)	322 (139)	0.37 (ns)	1.11 (ns)	1.18 (ns)
CRP	2,400 (2,270)	1,060 (360)	2,520 (2,590)	1,230 (309)	891 (239)	854 (119)	0.43 (ns)	0.61 (ns)	2.23 (ns)
Epiregulin	69,500 (16,300)	107,000 (15,000)	83,100 (14,600)	64,700 (15,900)	101,000 (13,000)	87,500 (39,300)	0.11 (ns)	4.59 (≤0.04)	0.046 (ns)
Fas	88.3 (31.0)	99.7 (29.4)	87.1 (11.1)	46.0 (21.3)	112 (76.0)	34.8 (18.3)	1.28 (ns)	2.50 (ns)	2.39 (ns)
Galectin-7	951 (35.8)	416 (302)	524 (269)	321 (101)	567 (182)	344 (43.8)	6.58 (≤0.02)	1.86 (ns)	6.20 (≤0.03)
gp130	3,770 (493)	6,010 (2,670)	6,430 (1,320)	6,590 (3,470)	3,170 (996)	4,550 (1,490)	3.39 (ns)	0.31 (ns)	0.44 (ns)
MMP-2	3,770 (436)	3,470 (1,100)	3,890 (424)	3,440 (831)	3,980 (1,200)	3,380 (970)	0.57 (ns)	0.030 (ns)	0.070 (ns)
MMP-3	14,300 (4,290)	18,800 (3,000)	16,600 (5,320)	21,400 (3,940)	15,500 (4,580)	15,000 (5,760)	2.23 (ns)	0.31 (ns)	0.12 (ns)
MMP-10	176 (47.2)	744 (205)	365 (10.6)	254 (80.9)	419 (119)	311 (46.7)	5.65 (≤0.02)	18.6 (≤0.00)	4.04 (ns)
PDGF-AA	52.2 (18.2)	33.6 (11.9)	35.1 (15.5)	38.2 (37.8)	34.5 (19.2)	31.4 (13.3)	0.19 (ns)	0.60 (ns)	0.32 (ns)
Persephin	79.0 (30.3)	96.1 (15.4)	130 (71.4)	125 (56.7)	155 (68.1)	67.4 (34.7)	2.62 (ns)	0.50 (ns)	0.35 (ns)
sFRP-3	226 (145)	220 (142)	255 (138)	106 (31.4)	106 (90.3)	99.3 (86.2)	0.060 (ns)	0.026 (ns)	5.93 (≤0.04)
Shh-N	139 (26.1)	132 (40.0)	143 (100)	75.1 (44.4)	70.4 (30.7)	147 (19.6)	0.86 (ns)	1.31 (ns)	2.84 (ns)
SLAM	1,730 (981)	1,740 (632)	950 (696)	1,340 (865)	445 (512)	1,030 (301)	1.49 (ns)	1.03 (ns)	2.62 (ns)
TGFb1	811 (229)	643 (150)	460 (334)	741 (241)	545 (129)	497 (208)	0.15 (ns)	2.66 (ns)	0.17 (ns)
TRANCE	662 (130)	486 (192)	433 (224)	257 (125)	550 (196)	239 (140)	2.80 (ns)	1.75 (ns)	4.84 (≤0.05)
TremL1	1,570 (1,090)	3,120 (2,100)	1,330 (1,330)	3,150 (2,390)	1,510 (405)	1,620 (362)	1.73 (ns)	0.67 (ns)	0.015 (ns)
TWEAK	180 (94.6)	265 (43.7)	270 (29.4)	160 (53.2)	284 (139)	236 (137)	0.13 (ns)	2.08 (ns)	0.070 (ns)
VEGF-B	235 (65.2)	187 (91.3)	115 (61.9)	57.0 (22.8)	252 (113)	134 (71.9)	4.30 (≤0.04)	2.56 (ns)	0.76 (ns)

**Supplementary Table S10: Y6FU Outcomes as a Function of Diet in Females.**				
**Metric**	**Breast**	**Cow Milk Formula**	**Soy Formula**	***P***
N	218	240	15	
IEP	8.3%	14%	6.7%	
Speech Therapy	7.3%	12%	6.7%	
Occupational Therapy	2.3%	2.9%	0%	
Help in School	4.1%	9.6%	6.7%	0.027[1]
Support in Classroom	0.92%	2.1%	6.7%	
Hay Fever or Respiratory Allergy	17%	25%	47%	0.0053[2]
Asthma	6.4%	12%	6.7%	
ADD or ADHD	0%	3.8%	0%	
Autism or Developmental Delay	0.46%	1.3%	0%	

[1]breast vs cow milk formula, Fisher exact test
[2]*P*=0.026 breast vs cow milk formula, *P*=0.0036 breast vs soy formula, *P*=0.064 cow milk versus soy formula by Chi square 3x2

**Supplementary Table S11: Y6FU Outcomes as a Function of Diet in Males.**				
**Metric**	**Breast**	**Cow Milk Formula**	**Soy Formula**	***P***
N	201	215	21	
IEP	12%	16%	24%	
Speech Therapy	11%	18%	24%	
Occupational Therapy	4.5%	7.0%	14%	
Help in School	7.0%	13%	24%	0.023[1,2]
Support in Classroom	4.5%	2.3%	19%	0.024[3,4]
Hay Fever or Respiratory Allergy	19%	24%	19%	
Asthma	10%	14%	19%	
ADD or ADHD	2.5%	6.5%	0%	
Autism or Developmental Delay	3.5%	6.1%	9.5%	

[1]Chi Square 3x2

[2]*P*=0.056 breast vs cow milk formula, *P*=0.0087 breast vs soy formula, *P*=0.15 cow milk vs soy formula by Chi square 2x2

[3]breast vs soy formula, Fisher exact test

[4]*P*=0.28 breast versus cow milk formula, *P*=0.0044 cow milk versus soy formula by Fisher exact test
